# *Zanthoxylum heitzii* Modulates Ferric Nitrilotriacetate-Dependent Oxidative Alterations in Four Vital Organs: An In Vitro Organoprotective Model

**DOI:** 10.1155/2017/6058150

**Published:** 2017-08-09

**Authors:** Jacques Joël Essogo, Bruno Moukette Moukette, Francine Nzufo Tankeu, Pauline Nanfack, Constant Anatole Pieme

**Affiliations:** Laboratory of Biochemistry, Department of Biochemistry and Physiological Sciences, Faculty of Medicine and Biomedical Sciences, University of Yaoundé I, P.O. Box 1364, Yaoundé, Cameroon

## Abstract

Ferric nitrilotriacetate (Fe-NTA) is a highly reactive compound used to induce degenerative disorders through oxidative stress (OS).* Zanthoxylum heitzii* (*Z. heitzii*) is a spice used as a medicinal plant to treat a variety of illnesses. This study investigated the ability of extracts from the leaves, fruits, roots, and barks of* Z. Heitzii* to inhibit Fe-NTA mediated oxidative damage in rats. The supernatant of rat liver homogenates was pretreated with the extracts for one hour before the induction of oxidative damage using a solution of Fe-NTA (400 mM). The activities of superoxide dismutase (SOD), catalase, and peroxidases were measured together with the marker of lipid peroxidation and the level of glutathione. The pretreated groups showed a significant increase in the activity of SOD, catalase, and peroxidases. The methanolic extract from the leaves of* Z. heitzii* (36.78 ± 3.30) and aqueous extract from the fruits (37.01 ± 2.52) showed the highest activities of SOD in the liver. The lowest concentration of MDA was found in the liver, and the glutathione was greater in the brain. Conclusively, these results suggest that* Z. heitzii* might be a chemoprotector which may be used in for prevention of distinct types of diseases induced by oxidative stress.

## 1. Introduction

Ferric nitrilotriacetate (Fe-NTA), a complexation of nitriloacetic acid with iron, has been described as a highly reactive compound and used in several studies to induce hyperglycemia, glycosuria, and both renal and liver carcinogenesis [1, 2]. Previous studies have demonstrated that the major pathway of Fe-NTA toxicity is through the generation of free radicals such as reactive oxygen species (ROS) [1, 3]. ROS cause alterations in the hepatic glutathione metabolizing enzyme, peroxidation of lipids, deterioration of proteins, and ultimately cellular and tissues injuries [1]. Previous research has demonstrated that a vast range of neurodegenerative diseases and the brain aging are correlated with oxidative stress [4–7]. The metabolism of the excitatory amino acid contributes significantly to the generation of ROS in the brain where they are particularly active. The presence of postmitotic cells such as glial cells which have a high predisposition to oxidative alteration amplifies the deleterious effects of ROS in the brain and may lead to the development of brain tumor, stroke, and other disorders [4]. Therefore, Fe-NTA produces an alteration of enzymatic processes and necrosis of hepatocytes in the liver whereas it causes an acute and subacute necrosis of the proximal tubule and renal DNA damage [1]. Fe-NTA has been used previously to induce a variety of disorders to investigate the biological properties of natural compounds against cancer, diabetes, and oxidative mediated toxicity in experimental settling both in vitro and in vitro [1, 3]. Recently, the mechanism of Fe-NTA induced toxicity has been described through its ability to cause an increase in lipid peroxidation and decrease in the concentration of enzymatic and nonenzymatic antioxidant molecules [1]. Epidemiological and biological data have both reported the beneficial impact of diet in the management of degenerative diseases [1]. Researchers have demonstrated at a molecular level that dietary components could inhibit the promotion or/and the propagation of cancer and inflammation [1].

In previous works, our group demonstrated the protective activities of different plants used as a spice in Cameroonian diet against iron-mediated oxidative damage on rat liver [8–10]. Our chemical analysis of those plant samples revealed a high amount in eugenol, apigenin, catechin, and quercetin in the studied extracts [11, 12]. We, therefore, hypothesized that the antioxidant content of a plant used in the diet could demonstrate some protective activities against diseases which involve oxidative stress in their pathogenesis [12–16].

*Zanthoxylum heitzii* (*Z. heitzii*) is a plant of the family Rutaceae which is widely distributed in the rain forest of Central Africa. Its fruits are used in Cameroon and the Democratic Republic of Congo as spices. Different parts of* Z. heitzii* are also used in African folk medicine to treat hypertension, gonorrhea, malaria, and cardiac disorders [15, 17]. Previous investigations have reported the antimicrobial activities of the bark of* Z. heitzii*; its fruits have also been reported to have beneficial effects against sickle cell diseases [15]. Chemical studies also had isolated and characterized a variety of active compounds from the bark of* Z. heitzii* [18]. Recently, meso-2,3-bis(3,4,5-trimethoxybenzyl)-1,4-butanediol and 4-acetoxy-2,3-bis(3,4,5-trimethoxybenzyl)-1-butanol, two alkaloids, and triterpenes were isolated from the bark of* Z. heitzii* and characterized [17]. Previously, its antiproliferative and antilarvicidal properties were reported [16, 19]. We investigated in the present study the in vitro chemoprotective effect of* Z. heitzii* against the toxicity of Fe-NTA on five homogenates from vital organs.

## 2. Material and Methods

### 2.1. Plant Material

**Figure 1:**
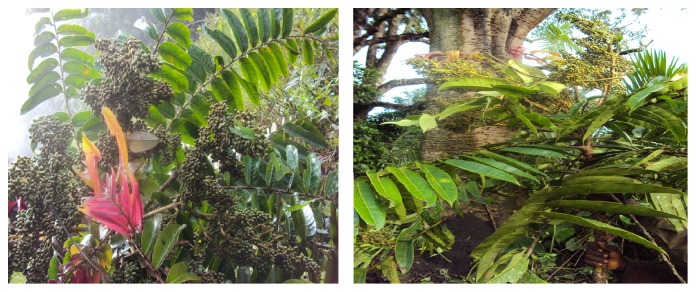
Leaves and fruits from* Zanthoxylum heitzii*.

The leaves, roots, bark, and fruit of* Z. heitzii* (Figure 1) were harvested on 03 June 2010 in West Cameroon. These samples have been identified at the national herbarium of Cameroon to the specimen number 1441/HNC. The samples were dried at room temperature and ground into powders which were kept in dry conditions.

### 2.2. Animals Description

Three adult albino rats of* Wistar *strain weighing between 150 and 200 g were used for this study. The animals were housed in polypropylene cages at the animal facility of the Faculty of Medicine and Biomedical Sciences of the University of Yaoundé I. They were maintained at room temperature with a natural light/dark cycle and had food and water ad libitum. The use of animal in this study was conducted after obtaining the approval of the Faculty of Medicine and Biomedical Sciences Ethical Committee.

### 2.3. Preparation of Tissue Homogenates

After being acclimatized for one week, the animals were sacrificed by decapitation, and their livers, kidneys, brains, and hearts were collected and weighed and put in phosphate buffer (0.1 mol/L, pH 7.8) supplemented with KCl (1.5%) in an ice bath. The organ homogenates were prepared by grinding the different samples in the phosphate buffer solution in the proportion of 10/100 (w/v). The mixture was then centrifuged at 5000 rpm for 30 min [9]. The supernatant was then collected and kept in a fridge at 4°C for further experiments [14].

### 2.4. Preparation of Ferric Nitrilotriacetate Solution

The oxidizing solution used in this study was prepared as previously described by Tankeu et al., 2016. The powders of FeCl_3_, 1.62 g (Sigma-Aldrich, Germany), and nitrilotriacetate, 7.64 g (Sigma-Aldrich, Germany), were dissolved in 100 mL of a solution of hydrochloric acid, 0.1 N (Sigma-Aldrich, Germany), for final concentrations of 200 mM and 400 mM, respectively. The obtained solution was then mixed to an H_2_O_2_, 200 mM 1 : 1 (v/v) (Fisher Scientific, USA). This oxidizing solution was used immediately after preparation [14, 20].

### 2.5. Treatment Procedure

The study was conducted as described in Table 1. After the incubation period, biochemical assays were performed on the different samples.

### 2.6. Biological Assays

#### 2.6.1. Determination of the Level of MDA

The concentration of MDA in the various rat homogenates was measured as previously described [21]. This assay relies on the reaction of the 2-thiobarbituric acid with malondialdehyde at 70°C. A single molecule of malondialdehyde complexes two molecules of 2-thiobarbituric acid Knoevenagel-type condensation to yield a chromophore with absorbance maximum at 532 nm. A volume of 2 mL of MDA working solution (Trichloroacetic acid (10 · 10^−3^ M) (Sigma-Aldrich, Germany) and 1 ml of 2-thiobarbituric acid (67 · 10^−3^ M) (Sigma-Aldrich, Germany) were added to a test tube containing 100 of the sample. The mixture was vortexed and incubated at 100°C for 15 min. Then the tubes were allowed to cool at room temperature and centrifugated at 3000 rpm for 5 min. The supernatant of each tube was collected and the OD were read at 532 nm [8].

#### 2.6.2. Determination of the Reduced Glutathione Level

The measurement of the concentration of reduced glutathione was realized using Ellman's method [22]. This assay is based on the reaction of glutathione with 5-5′-dithio-bis(2-nitrobenzoic acid) (DTNB) (Ellman's reagent) to produce a chromophore which can be measured at 412 nm [23]. A volume of 20 *µ*L of sample was added to a test tube containing 1 mL of Ellman's reagent (5-5′-dithio-bis(2-nitrobenzoic acid) in phosphate buffer pH 6.6, 0.1 M). The mixture was then homogenized and incubated at room temperature for three h. Then the OD were reassured at 412 nm, and the results were expressed in *µ*M/L using the formula OD = *ε* · *C* · *l*; *ε* = 13600 [14].

#### 2.6.3. Determination of the Total Protein Concentration

The total protein content of the mixture of the liver was measured according to the protein kit supplier methods (Human Kit-Hu102536, Boehringer, Ingelheim, Germany). This result was used to express the activities of the different enzymes per g of organs.

#### 2.6.4. Determination of the Superoxide Dismutase (SOD) Activity

The method described by Misra and Fridovich., 1979, was used. This method is based on the inhibition of autoxidation of epinephrine to its adrenochrome. A mixture of 580 *μ*L PBS, 200 *μ*L of each extract or standard, and 200 *μ*L of liver, kidney, kidney, and heart homogenate and 20 *μ*L of inducing solution was introduced into different test tubes and incubated at 37°C for 1 h. A volume (150 *µ*L) of each test solution was dispensed into tubes, and 500 *μ*L of carbonate buffer (pH 10.2; 0.3 M; pKa 10.3), 250 *μ*L of an EDTA solution (0.6 mM), and 350 *µ*L of distilled water were added. The mixture was homogenized, and 150 *μ*L of an epinephrine solution (4.5 mm) was added to initiate the reaction. Four other tubes were run simultaneously to serve as normal, negative, and positive controls in which the extract was replaced, respectively, by distilled water, oxidant, Vit C, and quercetin. The optical density was read after 30 seconds and 120 seconds at 480 nm. The following formula allowed the calculation of the SOD activity: SOD (unit/mg protein) SODunits/ml/mg protein (mg/ml × df), where df = dilution factor.

The SOD activity was thereafter expressed as Unit/min/mg of protein (UI/mg prot.)

#### 2.6.5. Determination of the Catalase Activity

The catalase activity of plant extracts on different homogenates was assessed according to a formerly described method [22] with some amendments. A volume (900 *µ*L) of phosphate buffer (0.01 M, pH 7) was introduced in tubes; thereafter, an aliquot (100 *µ*L) of the above test solutions was added to each tube then the mixture was vortexed. The addition of 400 *µ*L of a (200 mM) of Hydrogen peroxide solution to each tube started the reaction. After 1 min, 2000 *µ*L of acetic: dichromate solution (3 : 1) was added to stop the reaction. The mixture was boiled 10 min, and the absorbance was measured at 530 nm.

#### 2.6.6. Determination of the Peroxidase Activity

In different test tubes, 580 *μ*L of PBS (0.1 M; pH 7.4), 200 *μ*L of each plant extract or vit C and quercetin used as standards, 200 *μ*L of each homogenate (liver, heart, kidney, and brain), and 20 *μ*L oxidizing solution (HCl 0.1 M, FeCl_3_ 200 mM, NTA 400 mM, and H_2_O_2_ 200 mM) were introduced. The normal control and negative and positive controls were run simultaneously in the same conditions as described above. The mixtures were thereafter incubated at 37°C for 1 h. Then, 100 *μ*L of each of these mixtures was dispensed into new test tubes containing 900 *μ*L of PBS (0,01 M; pH 7). An aliquot of PBS 0,01 M, pH 6; pH 7 (320 *μ*L), hydrogen peroxide 0.05% (160 *μ*L), and pyrogallol solution 0.05% (320 *μ*L) were added to distilled water (210 *μ*L). A volume of 100 *μ*L from the above mixture was added thereafter. The reaction was mixed and incubated for at least 10 min, and the increase in absorbance at 420 nm was measured after 20 and 140 s using a spectrophotometer.

#### 2.6.7. Statistical Analysis

The different assays were conducted in triplicate, and the results were represented as mean ± SD. The program SPSS (Statistical Package for the Social Sciences) version 18.0 for Windows was used for the data analysis purpose. The statistical analysis was conducted using a one-way ANOVA (Analysis of variance) test followed by Kruskal-Wallis test, and Dunnett's multiple tests were used for the assessment of the Spearman rho correlation. *p* < 0.05 was considered as statistically significant.

## 3. Results

### 3.1. Effects of* Z. heitzii* on the Reduced Glutathione Level

**Figure 2:**
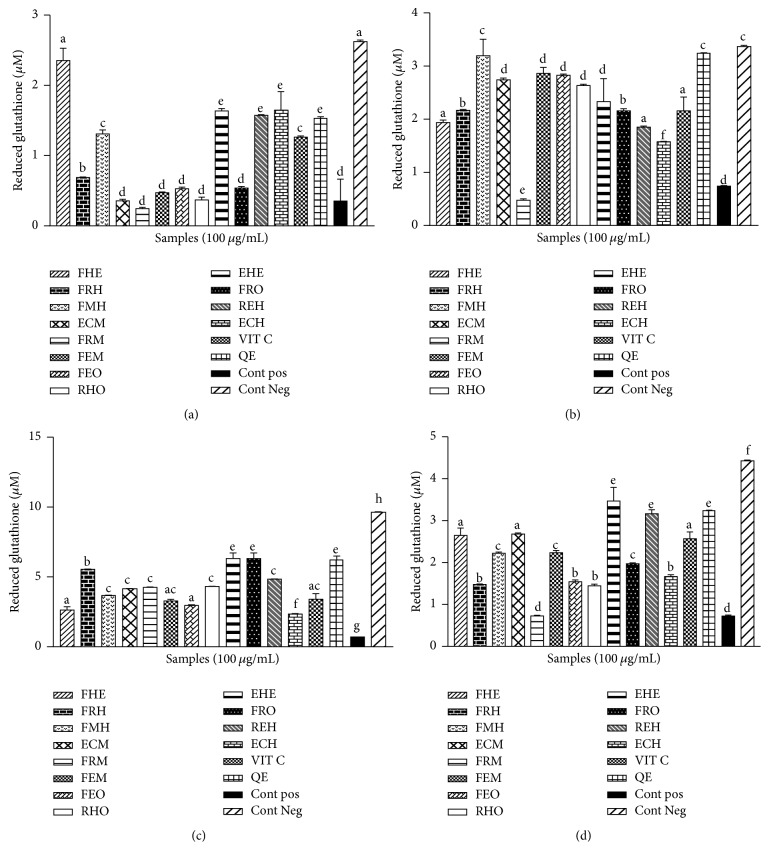
Effect of* Zanthoxylum heitzii* on the reduced glutathione level in rats. Values are expressed as mean ± SD of three replicates. In the same figure, the values affected with different letters are significantly different at *p* < 0.05. Concentration of glutathione in homogenates from (a) the brain, (b) the heart, (c) the liver, and (d) the kidney.

The exposure of the supernatant homogenates from the rat brain, liver, kidney, and heart to the Fe-NTA significantly (*p* < 0.05) decreased the glutathione levels. As represented in Figure 2, the treatment of the samples with the extracts from* Z. heitzii* has been beneficial for the glutathione concentration in the different samples. While a significant decrease in the level of glutathione was observed in the positive control 2.63 ± 0.26 *µ*M, as compared to the negative control 0.36 ± 0.16 *µ*M, an increase in the concentration was noted in the treated groups. In the brain, the ethanol/water extract from the leaves (FEH: 2.35 ± 0.05 *µ*M) showed the more elevated activity compared to the other samples (Figure 2(a)) while, in the heart, the methanol extract from the leaves (FMH: 3.19 ± 0.07 *µ*M) had the highest concentration (Figure 2(b)). These two samples had shown the higher activities of the tested extracts. Our results also showed a significant variation of the level of glutathione from one organ to another (Figures 2(c) and 2(d)).

### 3.2. Antilipoperoxidation Effect of* Z. heitzii*

**Figure 3:**
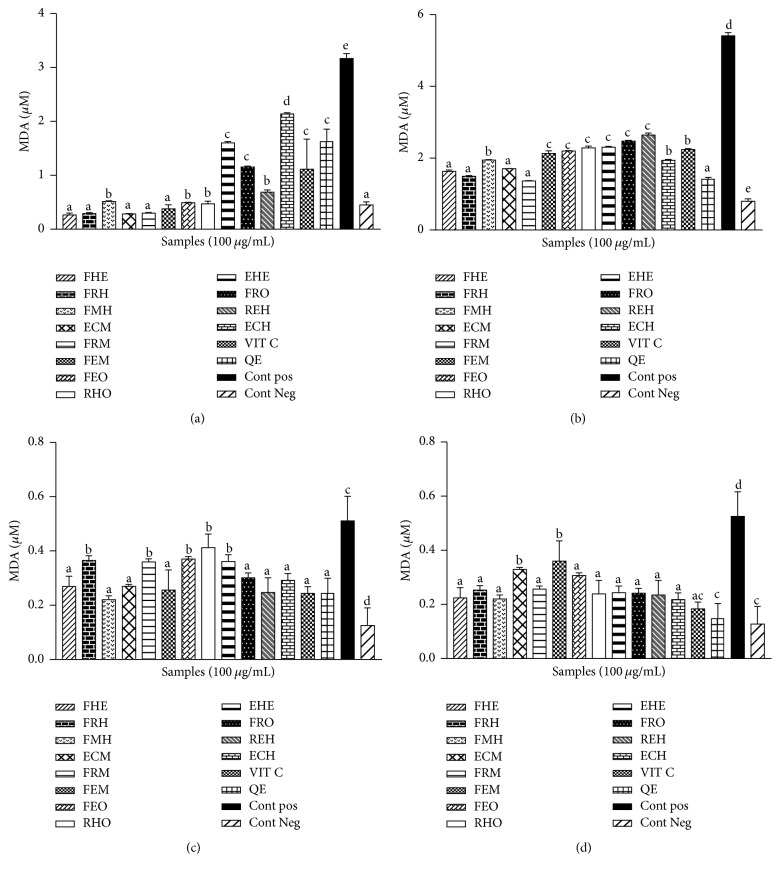
Effect of* Zanthoxylum heitzii* on the concentration of malondialdehyde (MDA) in rats. Values are expressed as mean ± SD of three replicates. In the same figure, the values affected with different letters are significantly different at *p* < 0.05. Concentration of malondialdehyde in homogenates from (a) the kidney, (b) the liver, (c) the heart, and (d) the brain.

The assessment of the lipoperoxidative effects of the Fe-NTA on the brain, the liver, the heart, and the kidney was realized by measuring the concentration of MDA. The results showed a significant (*p* < 0.05) increase in the level of MDA in the positive group as compared to the negative control (Figure 3). The pretreatment of the supernatant of the homogenates with the extracts from* Z. heitzii *has led to a decrease of the concentration of MDA in those groups. The results showed a higher antilipoperoxidative potential in the kidney (Figure 3(a)). The level of MDA in the liver ranged from 1.8 *µ*mol/L to 2.5 *μ*mol/L for all extracts (Figure 3(b)). The antilipoperoxidative potential was slightly higher in the liver compared to the kidney, the brain (Figure 3(c)), and the heart (Figure 3(d)).

### 3.3. Effect of* Z. heitzii* on the SOD Activity

**Figure 4:**
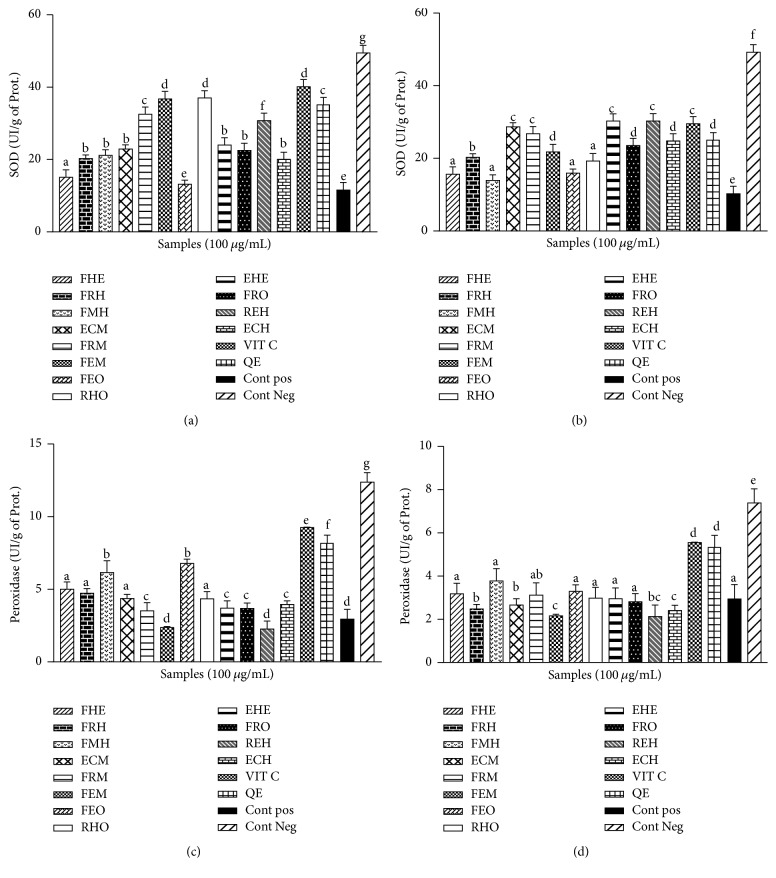
Effect of* Zanthoxylum heitzii* on superoxide dismutase (SOD) activity in rats. Values are expressed as mean ± SD of three replicates. In the same figure, the values affected with different letters are significantly different at *p* < 0.05. Concentration of superoxide dismutase in homogenates from (a) the liver, (b) the heart, (c) the kidney, and (d) the brain.

The chemoprotective potential of the extracts from* Z. Heitzii* was evaluated by the measurement of the activity of SOD. The exposure of the supernatant of different organ homogenates to the Fe-NTA has altered the activity of the SOD. The results showed a significant reduction of the SOD activity in the positive control group as compared to the negative control (Figure 4). The pretreatment of the samples with the extracts prevented the Fe-NTA deleterious effects. In the liver (Figure 4(a)) and the heart (Figure 4(b)), the activities of the SOD were significantly (*p* < 0.05) more elevated than those of the kidney (Figure 4(c)) and the brain (Figure 4(d)). The methanolic extract of the leaves (36.78 ± 3.30) and aqueous extract of the fruits (37.01 ± 2.52) have shown the highest activities of SOD in those samples with values similar to that of vitamin C.

### 3.4. Effect of* Z. heitzii* on the Catalase and Peroxidase Activities

**Figure 5:**
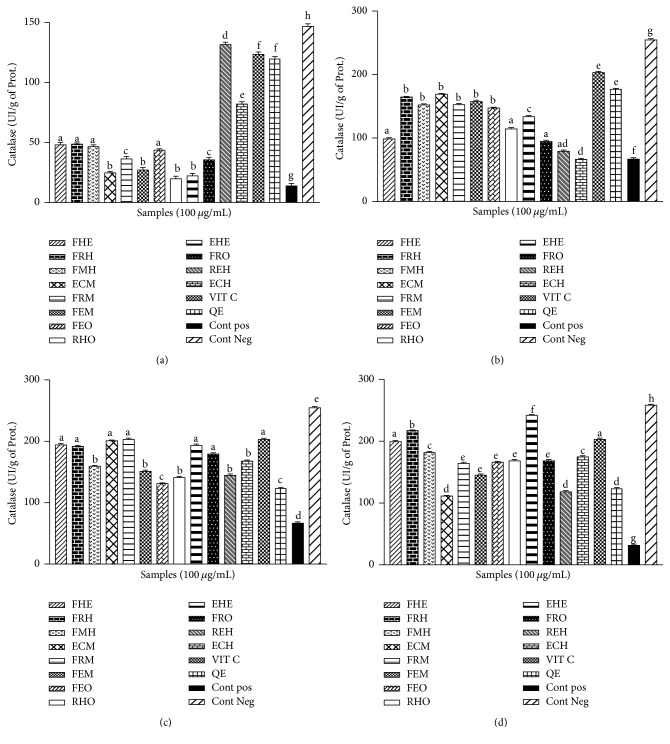
Effect of* Zanthoxylum heitzii* on catalase activity in rats. Values are expressed as mean ± SD of three replicates. In the same figure, the values affected with different letters are significantly different at *p* < 0.05. Concentration of catalase in homogenates from (a) the heart, (b) the liver, (c) the kidney, and (d) the brain.

**Figure 6:**
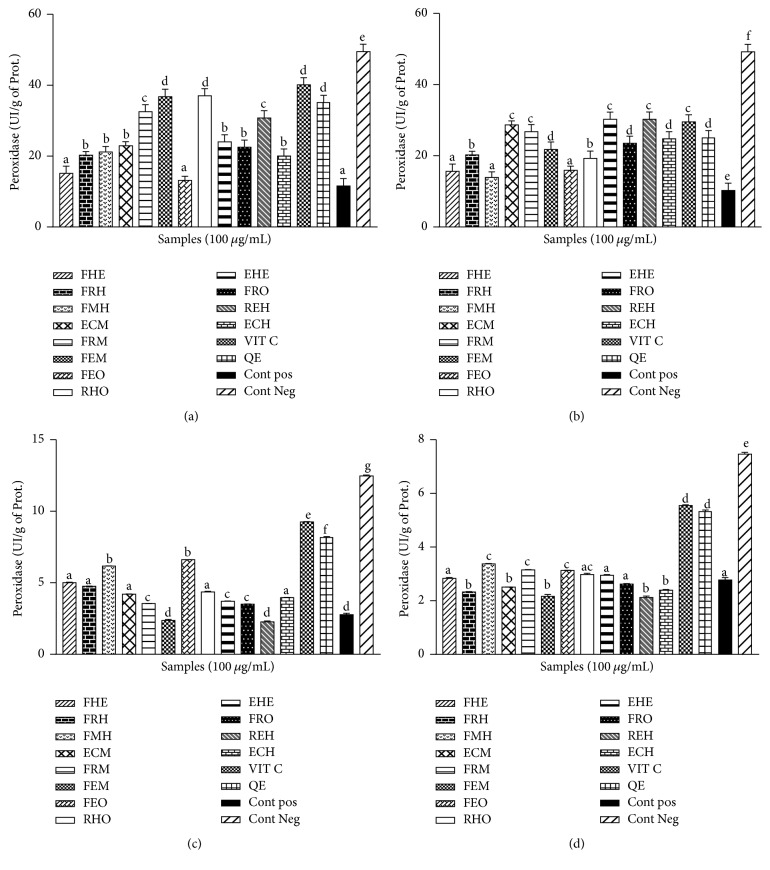
Effect of* Zanthoxylum heitzii* on peroxidase activity in rats. Values are expressed as mean ± SD of three replicates. In the same figure, the values affected with different letters are significantly different at *p* < 0.05. Concentration of peroxidase in homogenates from (a) the liver, (b) the heart, (c) the kidney, and (d) the brain.

The results obtained in the catalase and peroxidase activities showed a significant (*p* < 0.05) decrease of the activity of peroxidase and catalase in the presence of Fe-NTA. There was a significant (*p* < 0.05) reduction in the activities of catalase and peroxidase in the positive control group as compared to the negative control (Figures 5 and 6). The groups pretreated with the extract showed an increase in the level of these enzymes when compared to the positive control. The range values of the catalase activities in the liver (Figure 5(a)), kidney (Figure 5(c)), and brain (Figure 5(d)) are comprised between 125 IU/mg of prot. and 200 IU/mg of prot. and the ethanol/water extract from fruit presents the highest catalase activity as compared to the other organs. For the peroxidase concentration, the higher activity was observed in the supernatant of the heart homogenate (Figure 6(b)) and the liver (Figure 6(a)) ranging between 15 IU/mg of prot. and 40 IU/mg of prot. and the methanol extract of the leaves and the aqueous extract of the roots showed the highest peroxidase activities among the tested samples with peroxidase activities similar to that of vitamin C.

## 4. Discussion

The ability of a nitriloacetic acid to form a variety of water soluble complexes at a pH of 7 has been applied in several studies using its ion complex to induce experimental model of chronic diseases and intoxications [24]. Previous studies have revealed the ability of Fe-NTA to causes liver cancer, neurodegenerative alterations, and renal and hepatic injuries [25]. The Fe-NTA has been demonstrated to induce neoplastic transformation of a hepatic cell and to cause an increase in the level of free ion leading to an acute necrosis of the renal proximal tubule [24, 26]. Oxidative stress has been described as a primary feature of the mechanism underlying the physiological toxicity of Fe-NTA. This has been correlated with the ability of Fe-NTA to induce the production of hydroxyl radicals [27, 28]. Previously, it has been demonstrated that human diet contains many antimutagens and antioxidants. These compounds have been reported to play a significant role in the inhibition of the initiation and the promotion of some cancer [20]. Recently, researchers have been focused on the study of the chemoprotective and anticancer properties of a variety of antioxidants. This study is consistent with those findings and demonstrated the antioxidant and the chemopreventive effects of* Z. heitzii* on the brain, the liver, the heart, and the kidney.

The overproduction of ROS in the cell has been correlated to an increase of the alteration of macromolecules such as lipids [29]. This peroxidation of polyunsaturated fatty acids (PUFA) in turn induces a range of reaction leading to the production of lipid peroxide breakdown such as MDA [29]. The concentration of MDA in biological samples has been used in several studies as a marker of lipid peroxidation [28, 30]. In this study, we demonstrated a decrease in the level of MDA in the sample pretreated with the extracts from* Z. heitzii*. Our results corroborate previous authors who showed a variation of the oxidative level from one organ to the other, together with a modulation of the response in the presence of the extract [31]. These results demonstrate that the chemopromoting potential of Fe-NTA might be modulated by the cell environment. Our results corroborated previous findings which demonstrated the protective potential of probucol against the induction of renal and liver cancer by Fe-NTA in a rat model [29]. Phenolic compounds from natural plants have also been demonstrated to play a fundamental role in the biological potential of several nutraceuticals [32]. The mechanism underlying the protective potential of phenolic compounds has been explained by their ability to chelate iron and inhibit xanthine oxidase [31]. The ability of a phenolic compound to donate proton has been described as one of its more potent antilipoperoxidation properties [9]. This statement has been supported by previous work which reported the free radical scavenging potential of natural antioxidants [15]. The human body has a broad range of antioxidant molecules that help the cell to counterbalance the ROS [28]. That molecule can be of enzymatic origins such as the SOD, catalase, and peroxidase [10, 12]. This study has shown a significant variation of the activity of antioxidant enzymes from one organ to another corroborating previous authors who demonstrated the disparities in the antioxidant response between different vital organs [14]. The variation of the concentration of antioxidant enzymes in biological samples has been used in a diversity of study as a marker of oxidative stress [33]. We demonstrated in the present study an increase in the activity of SOD, catalase, and peroxidase in the groups pretreated with the extract of* Z. heitzii* compared to the control. This beneficial effect of* Z. heitzii* also varied from one organ to another supporting the hypothesis that the activity of this extract might be related to his ability to stimulate the production of antioxidant enzymes [34]. Our results corroborate previous authors who have demonstrated the role of polyphenolic compounds in the stimulation of the production of cellular Mn-SOD in the mitochondria [34]. The increase in the level of reduced glutathione in the different sample might be also supportive of the antioxidant properties of* Z. heitzii* by reducing ROS in the experimental medium [35]. In this study, a positive correlation has been observed between the decrease of oxidative damage and the increase in SOD, catalase, and peroxidase level. These findings support previous author results who demonstrated the beneficial effect of phenolic compounds such as resveratrol on a variety of cancer [35]. The results obtained from this study suggest that* Z. heitzii* might have some chemoprotective properties.

## 5. Conclusion

In the present study, we demonstrated the antioxidant and organoprotective effects of the extracts from* Z. heitzii* against oxidative stress induced by Fe-NTA in the homogenate of the brain, the kidney, the liver, and the heart. The extract has augmented the level of reduced glutathione and increased the activities of SOD, catalase, and peroxidase. These results suggest that* Z. heitzii* might be a very strong chemoprotector and may be used in the prevention of distinct types of diseases induced by oxidative stress.

## Figures and Tables

**Table 1:** Treatment protocol.

Reagents	Blank	Negative control	Positive control	Vitamin C	Samples
Phosphate buffer (0,1 m; pH 7,4) (*µ*l)	680	700	680	580	580
Plant extracts [100 *μ*g/ml] (*μ*l)	0	0	0	0	100
Vitamin C [1 mg/ml] (*µ*l)	0	0	0	100	0
Organ homogenates (*µ*l)	300	300	300	300	300
Homogenisation and incubation for 1 h at 37°C^*∗*^					
Prooxidant solution (*µ*l)	20	0	20	20	20
Homogenisation and incubation for 1 h at 37°C^*∗*^					

^*∗*^The samples were incubated in a hot water bath (37°C) and each of the columns represents a test group.

## Abbreviations

Vit C: Vitamin C
RNS: Reactive nitrogen species
ROS: Reactive oxygen species
FRAP: Ferric reducing antioxidant power
TBA: Thiobarbituric acid
H_2_O_2_: Hydrogen peroxide
CAT: Catalase
MeOH: Methanol
H_2_O: Distilled water
H_2_O/EtOH: Water/ethanol (70/30; V/V)
RMH: Roots MeOH
FEO: Leaves H_2_O
ECM: Barks MeOH
RHO: Roots H_2_O
ECH: Barks H_2_O/EtOH
FHE: Leaves H_2_O/EtOH
FEM: Leaves MeOH
EHE: Barks H_2_O
FRM: Fruits MeOH
RHE: Roots H_2_O/EtOH
FRO: Fruits H_2_O
FRH: Fruits H_2_O/EtOH
VIT C: Vitamin C
QE: Quercetin
Cont pos: Positive control
Cont neg: Negative control.

## Data Access

The authors confirm that the data supporting our findings are available in Constant Anatole Pieme's lab and are available if requested.
